# *In-silico* prediction of novel drug-target complex of nsp3 of CHIKV through molecular dynamic simulation

**DOI:** 10.1016/j.heliyon.2020.e04720

**Published:** 2020-08-24

**Authors:** Durgesh Kumar, Mahendra Kumar Meena, Kamlesh Kumari, Rajan Patel, Abhilash Jayaraj, Prashant Singh

**Affiliations:** a Department of Chemistry, Atma Ram Sanatan Dharma College, University of Delhi, New Delhi, India; b Department of Chemistry, University of Delhi, Delhi, India; c Deparment of Zoology, Deen Dayal Upadhyaya College, University of Delhi, New Delhi, India; d CIRBS, Jamia Millia Islamia, New Delhi, India; e SCFBio, Indian Institute of Technology, New Delhi, India

**Keywords:** Pharmaceutical chemistry, Theoretical chemistry, Multicomponent reactions (MCRs), nsp3 of CHIKV, Docking, DFT study, MD simulations

## Abstract

Literature reported that nsp3 of CHIKV is an important target for the designing of drug as it involves in the replication, survival etc. Herein, about eighteen million molecules available in the ZINC database are filtered against nsp3 using RASPD. Top five hit drug molecules were then taken from the total screened molecules (6988) from ZINC database. Then, a one pot-three components reaction is designed to get the pyrazolophthalazine and its formation was studied using DFT method. Authors created a library of 200 compounds using the product obtained in the reaction and filtered against nsp3 of CHIKV based on docking using iGEMDOCK, a computational tool. Authors have studied the best molecules after applying the the Lipinski's rule of five and bioactive score. Further, the authors took the best compound i.e. CMPD178 and performed the MD simulations and tdMD simulations with nsp3 protease using AMBER18. MD trajectories were studied to collect the information about the nsp3 of CHIKV with and without screened compound and then, MM-GBSA calculations were performed to calculate change in binding free energies for the formation of complex. The aim of the work is to find the potential candidate as promising inhibitor against nsp3 of CHIKV.

## 1 Introduction

Chikungunya Virus (CHIKV) causes chikungunya fever (CHIKF) and this virus spread through the biting of mosquito [1, 2]. It causes severe infection and the symptoms of CHIKF are high fever, polyarthralgia, myalgia etc. [3, 4, 5] Till date, there is no effective vaccine or drug for this disease available in the market, although few candidates as vaccine are under clinical trials [6]. Alphavirus is an enveloped viruses with a single stranded (+ss) RNA with non-structural proteins (nsP1234) and structural proteins, capsid, 3 envelope glycoproteins (E1, E2 and E3) and 6k peptide [7, 8, 9, 10, 11]. nsp3 of CHIKV is also known as macro-domain and have been initially obtained from databank [12]. Researchers reported baicalin as one of the potential drug molecule against the CHIKV based on binding affinity and π-π interaction between baicalin with TYR114 residue of nsP3 of CHIKV [13, 14]. Heterocyclic compounds have attracted the attention of the researchers due to biological potency in different aspects and they can be synthesized by number of steps as well one pot synthesis. Further, one pot synthesis or the multi-component reactions are preferred due to less time consumption in the synthesis as well less or no time is wasted in the purification of the compound [14, 15, 16, 17]. *In silico* methods are being explored by the researchers due to the efficiency and strategic approach. Computational tools are used to create a library and filtering them to get the biological potent compound against a receptor [18, 19, 20, 21, 22, 23, 24, 25, 26]. In this work, authors have designed a multi-component reaction (MCR) to produce pyrazolophthalazine via the one pot reaction between benzaldehyde, 2,3-dihydrophthalazine-1,4-dione and oxazolidine-2,4-dione (OZD) and its feasibility was studied through DFT method using Gaussian 09. Then, a library of 200 molecules was designed based on pyrazolophthalazine. Designed library was used for virtually screening against nsP3 of CHIKV, to get potential lead molecules based on minimum total binding energy, drug-likeness, and bioactivity score [27]. The filtered compounds were subjected to molecular docking using ParDOCK and their interaction profile was analyzed using DS visualizer, Pymol, Chimera. Further, temperature dependent molecular dynamic simulations (tdMD) and MM-GBSA of screened compound-nsp3 of CHIKV complex was performed to analyze the structural stability of the complex.

## 2 Materials and methods

### 2.1 Designed chemical reaction

Herein, CS ChemDraw was used to draw the chemical reaction using from benzaldehyde, 2,3-dihydrophthalazine-1,4-dione and oxazolidine-2, 4-dione (OZD) to get pyrazolophthalazine i.e., the product molecule. It was used to design drug library by changing alkyl group (from R_1_ to R_5_) in aryl of aldehyde and these molecules were considered to be potential drug molecule targeting nsp3 of CHIKV [15, 28].

Literature reported that the oxazolidine-2,4-diones are based on five member heterocyclic compounds and many biological activity are reported. They have shown promising role as aldose reductase inhibitors, hypoglycaemic and hypolipidemic agents.

### 2.2 Reaction mechanism through DFT

**Scheme 1:**
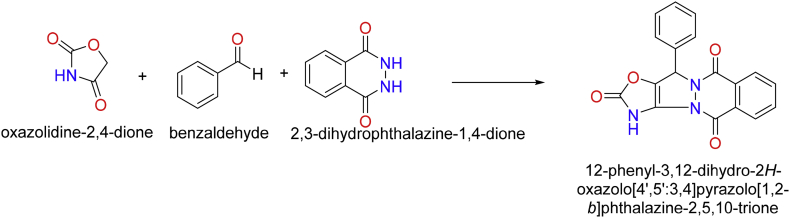
Synthesis of pyrazolophthalazine via the one pot three component reaction between benzaldehyde, 2,3-dihydrophthalazine-1,4-dione and oxazolidine-2, 4-dione (OZD).

**Scheme 2:**
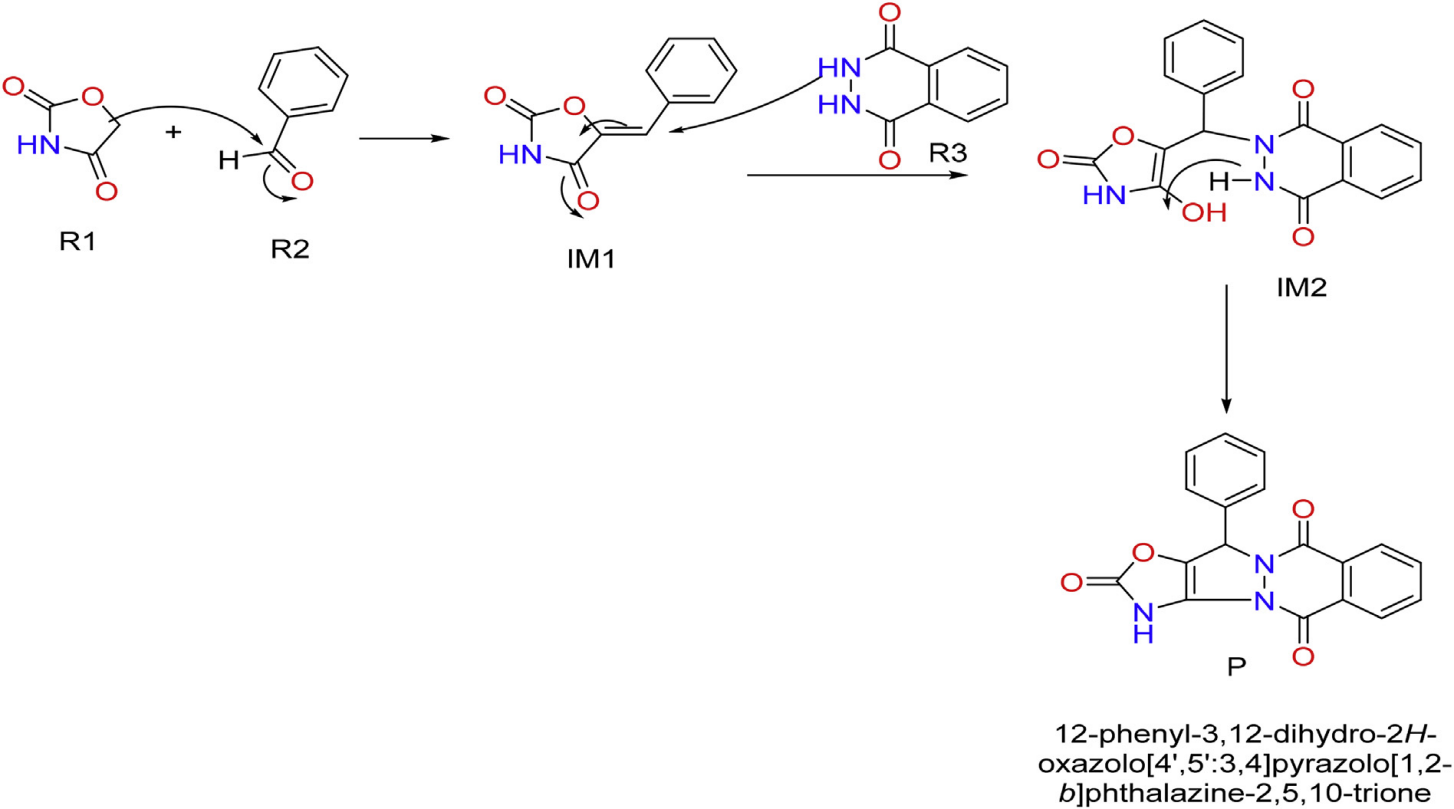
The proposed mechanism for the formation of pyrazolophthalazine via one pot three component synthesis.

A novel MCRs for the formation of novel pyrazolophthalazine molecules through a reaction between oxazolidine-2,4-dione, benzaldehyde and 2,3-dihydrophthalazine-1,4-dione is designed as shown in Scheme 1 and was studied using DFT. It is a proposed mechanism for the reaction shown in Scheme 2. Initially, there is a reaction between R1 i.e. OZD, has active methylene group and R2 i.e. benzaldehyde, carbonyl group. The reaction will give an unsaturated compound (IM1) with an elimination of water molecule via knoevangel reaction. Further, IM1 reacts with R3 i.e. 2,3-dihydrophthalazine-1,4-dione to give IM2. Herein, the lone pair present on nitrogen of 2,3-dihydrophthalazine-1,4-dione (nucleophilic site) attack on the unsaturated carbon (electrophilic site). It is aza-michael addition followed by the rearrangement to IM2. Further, IM2 loose a molecule of water and cyclization occurs to give P, the product of interest. The adduct formation is justified based on energy diagram using B3LYP/6-311G∗method [29, 30]. The following parameters of global reacting indices were calculated from reactant to product such as total energy (E), E_HOMO_, E_LUMO_ & LUMO-HOMO energy gap (ΔE) are calculated [31, 32]. The proposed mechanism of MCRs was studied using density functional theory (DFT) calculation as in Scheme 2.

### 2.3 Drug library and target preparation

A library of 200 molecules was created via different susbtitutents on pyrazolophthalazine using CS ChemDraw as in Table 1 [33, 34]. In designing, only aromatic aldehydes are varied to a library of the new compounds which may have better potency against the nsp3 of CHIKV. In this designed molecules only aromatic aldehydes have been varied by changing alkyl group R_1_ to R_5_. Library of designed molecules were used to screen against nsp3 of CHIKV through iGEMDOCK. Authors have been taken best five molecules on the basis of minimum total binding energy. Further, screening of best five molecules was filtered through ADMET properties. The crystal structure of nsP3 of CHIKV was obtained from the RCSB protein data bank (PDB ID: 3GPO) in complex with ADP-ribose at resolutions of 1.9 Ǻ respectively. The removal of extra atoms like water, missing atoms and added explicit hydrogen in the both model (ligand & target protein) was done using Pymol (BIOvIA 2015) and UCSF Chimera-1.13.1 software [35].

**Table 1:** The designed library of 200 molecules based on pyrazolophthalazine by changing alkyl group i.e. R_1_ to R_5_.

Parent Compound	CMPD	R_1_	R_2_	R_4_	CMPD	R1	R2	R_4_
	1	-NH_2_	-Br	-Br	12	-OH	-OCH_3_	-Cl
	2	-OCH_3_	-Br	-Br	13	-OH	-NO_2_	-OCH_3_
	3	-OH	-Br	-Br	14	-OH	-CH_3_	-I
	4	-OH	-Br	-Cl	15	-OH	-CH_3_	-Cl
	5	-OH	-Br	-NO_2_	16	-OH	-Cl	-F
	6	-OCH_3_	-OCH_3_	-Br	17	-OH	-CH_3_	-F
	7	-OH	-NO_2_	-Br	18	-F	-F	-F
	8	-OH	-OCH_3_	-Br	19	-OH	-F	-F
	9	-OH	-Cl	-Cl	20	-OH	-F	-Br
	10	-Cl	-Cl	-Cl	21	-F	-Cl	-CF_3_
	11	-OCH_3_	-OCH_3_	-Cl				
Parent Compound	CMPD	R_1_	R_3_	R_4_	CMPD	R_1_	R_3_	R_4_
	22	-I	-CH_3_	-CH_3_	27	-F	-F	-OCH_3_
	23	-I	-OH	-CH_3_	28	-Br	-OCH_3_	-OH
	24	-Br	-F	-Br	29	-Br	-OCH_3_	-OCH_3_
	25	-F	-OCH_3_	-CH_3_	30	-OCH_3_	-OCH_3_	-Br
	26	-F	-F	-F				
Parent Compound	CMPD	R_1_	R_4_	R_5_	CMPD	R_1_	R_4_	R_5_
	31	-NO_2_	-OCH_3_	-OCH_3_	36	-Cl	-Cl	-Cl
	32	-F	-Cl	-F	37	-Br	-CH_3_	-OH
	33	-Cl	-OCH_3_	-F				
	34	-Cl	-CH_3_	-F				
	35	-F	-F	-Cl				
Parent Compound	CMPD	R_1_	R_3_	R_5_				
	38	-F	-OCH_3_	-F				
	39	-F	-Cl	-F				
	40	-F	-CN	-F				
	41	-F	-Br	-F				
Parent Compound	CMPD	R_2_	R_3_	R_4_	CMPD	R_2_	R_3_	R_4_
	42	-Br	-OH	-Br	48	-Cl	-OH	-F
	43	-Br	-OCH_3_	-OCH_3_	49	-Cl	-OH	-OCH_3_
	44	-OCH_3_	-OH	-I	50	-Cl	-OCH_3_	-OCH_3_
	45	-OCH_3_	-OCH_3_	-I	51	-Br	-OH	-OCH_3_
	46	-F	-F	-F	52	-Br	-OH	-Cl
	47	-F	-OH	-OCH_3_				
Parent Compound	CMPD	R_2_	R_3_		CMPD	R_2_	R_3_	
	53	-OCH_3_	-OCH_2_CH_2_Br		67	-F	-Br	
	54	-Br	-CH_3_		68	-OCH_3_	-F	
	55	-NO_2_	-Br		69	-NO_2_	-F	
	56	-Br	-OCH_3_		70	-Cl	-F	
	57	-Br	-OH		71	-CH_3_	-F	
	58	-OH	-Cl		72	-CN	-F	
	59	-NO_2_	-Cl		73	-Br	-F	
	60	-Cl	-CH_3_		74	-OH	-OCF_2_H	
	61	-OCH_3_	I		75	-CF_3_	-F	
	62	-OCH_3_	-F		76	-CF_3_	-Cl	
	63	-F	-F		77	-CF_3_	-CF_3_	
	64	-F	-Cl		78	-Cl	-OH	
	65	-F	-CH_3_		79	-Cl	-OCH_3_	
	66	-F	-CN		80	-Cl	-Cl	
Parent Compound	CMPD	R_1_	R_4_		CMPD	R_1_	R_4_	
	81	-Br	-Br		93	-F	-NO_2_	
	82	-Br	-OCH_3_		94	-F	-F	
	83	-Br	-OH		95	-F	-Cl	
	84	-OCH_3_	-Br		96	-F	-Br	
	85	-OH	-Br		97	-F	-CF_3_	
	86	-NO_2_	-OH		98	-Cl	-CF_3_	
	87	-I	-OCH_3_		99	-CF_3_	-CF_3_	
	88	-OH	-F		100	-OH	-Cl	
	89	-OCH_3_	-F		101	-NO_2_	-Cl	
	90	-CH_3_	-F		102	-Cl	-NO_2_	
	91	-Br	-F		103	-Cl	-Cl	
	92	-F	-OCH_3_					
Parent Compound	CMPD	R_1_	R_3_		CMPD	R_1_	R_3_	
	104	-NO_2_	-NO_2_		114	-NO_2_	-Cl	
	105	-Cl	-F		115	-Cl	-OH	
	106	-CH_3_	-F		116	-Cl	-Cl	
	107	-F	-OCH_3_		117	-Cl	-CH_3_	
	108	-F	-F		118	-OH	-Br	
	109	-F	-Br		119	-OCH_3_	-Br	
	110	-NO_2_	-CF_3_		120	-NO_2_	-Br	
	111	-F	-CF_3_		121	-Br	-OCH_3_	
	112	-CF_3_	-F		122	-Br	-Cl	
	113	-OCH_3_	-Cl		123	-Br	-CH_3_	
Parent Compound	CMPD	R_2_	R_4_		CMPD	R_2_	R_4_	
	124	-OH	-NO_2_		128	-Cl	-Cl	
	125	-F	-F		129	-Br	-NO_2_	
	126	-CF_3_	-F		130	-Br	-Cl	
	127	-CF_3_	-CF_3_		131	-Br	-Br	
Parent Compound	CMPD	R_2_	R_5_		CMPD	R_2_	R_5_	
	132	-CN	-OCH_3_		136	-OH	-Cl	
	133	-I	-OH		137	-NO_2_	-Cl	
	134	-I	-OCH_3_		138	-Cl	-Cl	
	135	-F	-I		139	-CH_3_	-Cl	
Parent Compound	CMPD	R_1_	R_2_		CMPD	R_1_	R_2_	
	140	-OH	-NO_2_		146	-F	-CF_3_	
	141	-F	-OCH_3_		147	-Cl	-CF_3_	
	142	-F	-F		148	-OH	-OH	
	143	-F	-Cl		149	-OH	-OCH_3_	
	144	-OH	-F		150	-OH	-Br	
	145	-CH_3_	-F		151	-Br	-OH	
Parent Compound	CMPD	R_1_	R_5_		CMPD	R_1_	R_5_	
	152	-NO_2_	-NO_2_		156	-F	-Cl	
	153	-I	-F		157	-F	-CH_3_	
	154	-F	-OCH_3_		158	-Br	-F	
	155	-F	-F		159	-F	-CF_3_	
Parent Compound	CMPD	R_1_				CMPD	R_1_	
	160	-CN				164	-Cl	
	161	-I				165	-Br	
	162	-F				166	-H	
	163	-CF_3_				167	-NO_2_	
Parent Compound	CMPD	R_3_				CMPD	R_3_	
	168	-CN				173	-SCF_3_	
	169	-I				174	-OCF_3_	
	170	-F				175	-CF_3_	
	171	-OCF_2_H				176	-Cl	
	172	-OCF_2_CF_2_H				177	-Br	
Parent Compound	CMPD	R_2_				CMPD	R_2_	
	178	-NO_2_				184	-OCF_2_CF_2_H	
	179	-CN				185	-OCF_3_	
	180	-I				186	-CF_3_	
	181	-F				187	-Cl	
	182	-OCF_2_H				188	-NH_2_	
	183	-CF_2_H				189	-Br	
Parent Compound	CMPD	R_1_	R_2_	R_3_				
	190	-I	-OH	-OCH_3_				
	191	-F	-F	-F				
	192	-Cl	-OH	-OCH_3_				
	193	-Cl	-OCH_3_	-CH_3_				
	194	-Br	-OH	-OCH_3_				
Parent Compound	CMPD	R_1_	R_2_	R_3_	R_4_			
	195	-OH	-Br	-OCH_3_	-Br			
	196	-Br	-OCH_3_	-OCH_3_	-OCH_3_			
Parent Compound	CMPD	R_1_	R_2_	R_4_	R_5_			
	197	-F	-F	-F	-F			
	198	-Br	-Br	-OCH_3_	-OH			
	199	-Br	-F	-Cl	-I			
	200	-F	-Cl	-F	-Cl			

### 2.4 Virtual screening

RASPD is used for preliminary screening of potential molecules from Zinc database based on minimum binding free energy. This is very fast protocol for accurate prediction of hit candidates for any target protein. In this way, authors screened 6988 drug molecules from Zinc database based on binding affinity range of -14.8 to 10.0 kcal/mol, but top five drug molecules were selected from screened molecules on the basis of molecular weight (MW < 500) with minimum binding free energy for molecular docking and simulations [36]. In this protocol, **Method A** (Protein-Ligand Complex) was used to estimation of binding free energy and these molecules are listed in Table 2.

**Table 2:** Top five hit drug molecules with their binding energy against nsp3 protease of CHIKV screened from Zinc database using RASPD web server.

-13.0 ZINC13943005	-12.9 ZINC08680620	-12.8 ZINC00793735
-12.9 ZINC11790332	-12.8 ZINC01158015	

Screening is a method to design the drug in short span of time. It is used to investigate potential molecules against nsp3 of CHIKV. The purpose of this method is used to predict a best pose of molecule and it was selected best ligand conformations based on pose and their binding free energy [37]. The designed library of 200 molecules and screened top five molecules from RASPD were screened against nsP3 of CHIKV using iGEMDOCK software [38]. In this, top five best molecules from the designed library based on binding energy were taken and on other side, screened molecules from RASPD are ignored due to high binding energy in comparison of designed best molecules. iGEMDOCK computes a ligand conformation and orientation relative to the active site of target protein based on GA and summarized results in term of minimum total binding energy of the complex in Tables 4 and 5 [39].

### 2.5 Biological parameters

The bioactive properties like TPSA, chemical structure, LogP and Lipinski's “Rule of Five” value using Molinspiration were calculated [40, 41]. Several other biological parameters of best five compounds were calculated using Swiss ADME as in Tables 6 and 7. Thus, the absorption (% ABS) was calculated by given equation according to the method [42].

**%ABS** = **109** – **[0.345** × **topological polar surface area (TPSA)]**

### 2.6 Toxicity prediction

Herein, authors used GUSAR, a webserver to predict LD_50_ values for rats with four types of administration like Oral, Intravenous, Intraperitoneal, Subcutaneous and Inhalation. The acute toxicity of CMPD178, 53, 140, 173 & 124 has been calculated for screened molecules. These results were obtained through GUSAR for prediction of rat acute toxicity and acute rodent toxicity with four type of administration are mentioned in Table 8. The acute rate toxicity end-points are based on the log10 representation of LD_50_ value (mg/kg) for the rats [43].

### 2.7 Rigid docking

ParDOCK is an automated web server for rigid docking was used to determine the binding modes of compounds in the receptor [44].

E = ∑Eel + EvdW + Ehpb

E is the total energy; Eel is energy due to electrostatic interactions; EvdW is due to van der Waals interactions; Ehpb is the due to hydrophobic interactions.

### 2.8 Molecular dynamics simulations

The docking result was further validated using the molecular dynamics (MD) simulations method. MD simulations is used to predict the binding of compounds to the target protein and study the changes in binding strength with change in the temperature [45]. MD simulations, an important approach is to study the physical movements of the atoms of the receptor in presence and absence of the compound for a known time [46]. Three dimensional structure of the CMPD178 was drawn using Marvin sketch and its optimization is done with the Gaussian 09 on applying B3LYP/6-31G(d) basis [47, 48]. Further, other parameters of screened CMPD178 for the MD simulations were produced using antechamber module of AMBER suite molecular dynamics software utilizing Generalized Amber Force Field (GAFF) [49]. The input files were used to run by xleap command using Amber ff14SB force field for the created parameter and coordinate files. Subsequently, solvate box TIP3P 10.0 was added with a 10 Å buffering distance [50]. During thermalization, initial velocities were produced based on the Maxwell-Boltzmann distribution with a temperature of 300 K and constant volume (ntb = 1) for 20 ps (nstlim = 10000 × dt = 0.002) simulation time. Further, the receptor or its complex was equilibrated at 300 K and 1 bar using the Berendsen thermostat for constant pressure (ntp = 1) for another 500 ps simulation time. Once the equilibrium is reached, MD simulations were performed for 100 ns [51]. Different trajectories based on MD simulations were analyzed [52]. Authors also checked the RMSD value by the variable temperature (non-isothermally) of drug-target complex at 10 ns and according to David and Konard approximation and this approximation is said to be temperature dependent MD simulations. In tdMD simulation for 10 ns, the input file (temp 300K to 400k) was used to set print energy output files every 500 steps (ntwx&ntwr = 500) and save coordinates every 500 (ntwx = 500) as in amber input.

### 2.9 MM-GBSA method

MD simulations trajectories of complex system were used to determined relative change in binding free energy according to the MM-GBSA method [53, 54, 55, 56]. In order to calculate binding free energies like for CMPD178-nsP3 of CHIKV, nsP3 of CHIKV and CMPD178 was calculated for high accuracy results [23, 57]. The binding free energy (**ΔG**_bind_) of the drug-target complex is calculated on the given Eq. (1).

(1) **ΔG**_**bind**_ = **ΔG**_**bind**_, _**Vacuum**_ + **(ΔG**_**Solv,d-t**_ - **ΔG**_**Solv, d**_ - **ΔG**_**Solv, t**_**)**

Where, **ΔG**_bind_ and **ΔG**_bind_, _Vacuum_ are the free energy difference between the bound and unbound forms of a complex in solvated and vacuum respectively. **ΔG**_Solv,d-t,_ **ΔG**_Solv,d_ and **ΔG**_Solv,t_ represented the change in free energy between the solvated and vacuum states of a CMPD178, nsp3 of CHIKV and CMPD178-nsp3 of CHIKV complex. The change in solvation free energy from different systems are calculated by given Eqs. (2), (3), (4), and (5).

(2) **ΔG**_**gas**_**= G**_**gas**_**(d-t)** – **G**_**gas**_**(d) – G**_**gas**_**(t)**

(3) **ΔG**_**solv**_ = **G**_**solv**_**(d-t)** – **G**_**solv**_**(d) – G**_**solv**_**(t)**

(4) **G = [{E**_**MM**_**}** + **{G**_**Solv(polar + nonpolar)**_**}** – **T{S**_**MM**_**}]**

(5) **E**_**MM**_**= E**_**Int**_ + **E**_**el**_ + **E**_**vdW**_

E_MM_ - MM energy; E_int_ - internal energy; E_el_ - electrostatic energy and E_vdW_ - energy due to van der Waals interactions.

### 2.10 DFT studies of the top five hit screened drug molecules

Bonding orbital calculations were performed by full NBO program as executed in the Gaussian 09 [58]. Different physiochemical descriptors like electronic chemical potential (μ), global electronegativity (χ) and chemical hardness (η) global electrophilicity (ω) can be calculated from energies of HOMO and LUMO (Domingo et al., 2016) as in Eqs. (6), (7), (8), and (9) as follows.

(6) **μ = (E**_**HOMO**_**+ E**_**LUMO**_**) / 2**

(7) **χ = − (E**_**HOMO**_**+ E**_**LUMO**_**) / 2**

(8) **η = (E**_**LUMO**_**− E**_**HOMO**_**) / 2**

(9) *ω***= μ**^**2**^**/ 2η**

## 3 Results and discussion

Authors have designed the chemical reaction for the synthesis of biologically potent pyrazolophthalazine as in Scheme 1 and it is considered as a novel compound to target nsP3 of CHIKV. Initially, there is a reaction between R1 i.e. OZD, has active methylene group and R2 i.e. caronyl of aromatic aldehyde. The reaction will give an unsaturated compound (IM1) with an elimination of water molecule via knoevangel reaction. Further, IM1 reacts with R3 i.e. 2,3-dihydrophthalazine-1,4-dione to give IM2. Herein, the lone pair present on nitrogen of 2,3-dihydrophthalazine-1,4-dione (nucleophilic site) attack on the unsaturated carbon (electrophilic site). It is aza-michael addition followed by the rearrangement to IM2. Further, IM2 loose a molecule of water and cyclization occurs to give P, the product of interest as in Scheme 2.

**Figure 1:**
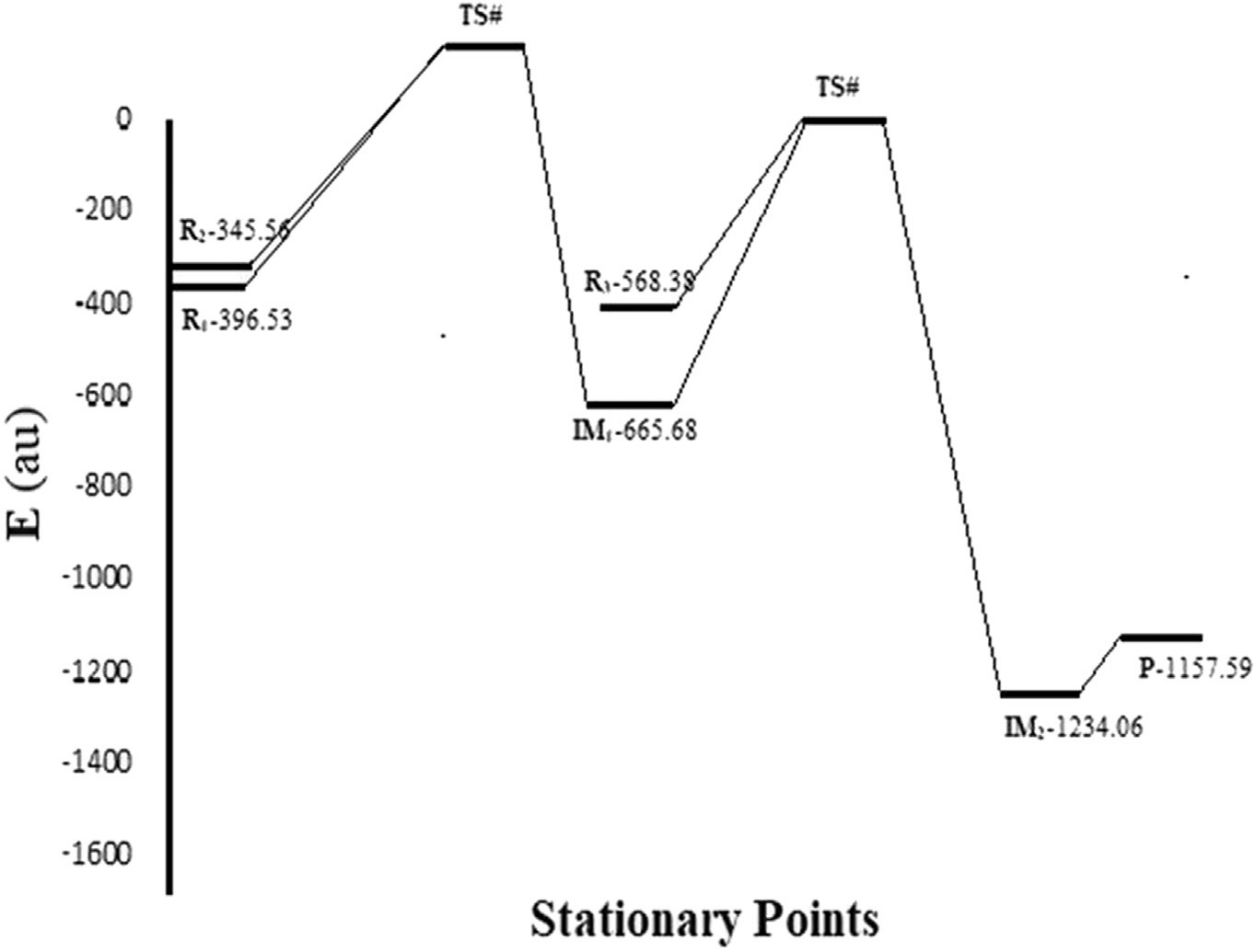
The energy profile diagram of product formation using B3LYP/6-311G∗ basis set as in Scheme 1 through DFT method.

Energy level (HOMO & LUMO) of reactant, intermediate and the product are determined. The energy differences between the orbital energies are shown in Table 3. The energy values of HOMO orbital and LUMO orbital of product molecules were lying at an energy value of -0.22767 eV and -0.09002 eV respectively. The LUMO-HOMO energy gap was obtained at -0.13765 eV in the isolated gas molecular calculations. If LUMO-HOMO energy gap is higher implies the kinetic energy is higher and high chemical reactivity [59] (see Figure 1).

**Table 3:** Energies of HOMO, LUMO, E and LUMO-HOMO (ΔE) for the formation product through intermediate from R1 to P.

S. No.	HOMO	LUMO	E_HOMO_	E_LUMO_	ΔE	E (au)
Reactant 1 (R1)			-0.30079	-0.05767	-0.24312	-396.53
Reactant (R2)			-0.26531	-0.07827	-0.18704	-345.56
Intermediate 1 (IM1)			-0.25135	-0.10038	-0.15097	-665.68
Reactant 3 (R3)			-0.24157	-0.07293	-0.16864	-568.38
Intermediate 2 (IM2)			-0.22644	-0.08405	-0.14239	-1234.06
Product (P)			-0.22767	-0.09002	-0.13765	-1157.59

### 3.1 Virtual screening

iGEMDOCK is used for the virtual screening of the compounds against the target protein [60, 61]. The details of the binding energy of the designed compounds against the nsp3 of CHIKV is given in Table 4 and screened molecules by RASPD from zinc data base against nsp3 of CHIKV is in Table 5. Then, the top five compounds are taken based on the least binding energy of the complex system for molecular docking.

**Table 4:** Total binding energy of designed 200 drug molecules against nsp3 of CHIKV.

CMPD	B.E.	CMPD	B.E.	CMPD	B.E.	CMPD	B.E.	CMPD	B.E.
178	-141.759	69	-121.974	75	-116.869	198	-111.738	67	-106.945
53	-139.509	18	-121.926	17	-116.8	64	-110.965	19	-106.749
140	-139.275	154	-121.748	107	-116.675	73	-110.923	42	-106.569
173	-136.842	56	-121.667	186	-116.559	96	-110.899	46	-106.558
124	-136.113	5	-120.851	168	-116.496	71	-110.808	175	-106.503
13	-132.881	98	-120.575	182	-116.491	139	-110.751	137	-106.088
74	-132.417	146	-120.573	166	-116.467	60	-110.695	150	-105.941
115	-130.88	16	-120.57	23	-116.346	189	-110.671	135	-105.854
121	-130.452	106	-120.285	50	-115.985	83	-110.44	90	-105.762
160	-130.423	188	-120.239	183	-115.929	190	-110.419	116	-105.725
51	-129.66	174	-120.055	57	-115.838	164	-110.337	119	-105.655
31	-129.647	152	-120.049	68	-115.828	100	-110.207	26	-105.541
193	-128.963	34	-119.877	24	-115.789	58	-110.206	78	-105.53
200	-128.837	171	-119.819	114	-115.518	80	-110.194	170	-105.439
110	-128.406	142	-119.745	52	-115.482	63	-110.096	117	-105.259
87	-128.37	49	-119.738	28	-115.388	89	-109.86	97	-105.148
122	-128.362	55	-119.723	129	-115.193	40	-109.853	138	-105.134
33	-127.291	185	-119.601	112	-115.123	103	-109.834	15	-105.008
194	-127.244	125	-119.559	6	-114.839	82	-109.633	134	-105.008
86	-126.539	76	-119.473	44	-114.762	133	-109.517	91	-104.877
77	-126.321	149	-119.406	22	-114.685	84	-109.509	163	-104.771
157	-125.865	3	-119.294	128	-114.633	197	-109.287	187	-104.321
12	-125.123	72	-118.966	36	-114.542	95	-109.264	54	-103.971
79	-124.857	127	-118.45	102	-114.39	159	-108.947	177	-103.82
27	-124.363	7	-118.438	48	-114.281	37	-108.851	66	-103.649
93	-124.303	153	-118.437	47	-114.222	143	-108.784	126	-103.172
196	-124.247	123	-118.359	108	-114.08	11	-108.679	176	-103.107
132	-124.175	118	-118.04	105	-113.907	136	-108.554	130	-103.049
161	-124.113	192	-118.029	30	-113.873	45	-108.519	131	-102.591
147	-123.895	85	-117.944	101	-113.865	144	-108.331	165	-102.427
1	-123.74	94	-117.838	70	-113.835	162	-108.123	156	-101.901
20	-123.552	151	-117.801	39	-113.824	111	-108.118	148	-101.349
88	-123.468	155	-117.374	10	-113.783	8	-107.838	169	-101.233
43	-123.235	61	-117.358	59	-113.383	99	-107.805	113	-99.1969
104	-122.971	184	-117.277	65	-113.151	181	-107.777	167	-98.8528
199	-122.674	32	-117.271	14	-112.663	2	-107.68	4	-98.1906
21	-122.624	25	-117.224	41	-112.097	145	-107.638	158	-97.6982
9	-122.441	38	-117.079	195	-112.026	62	-107.569	81	-97.2465
29	-122.388	179	-117.06	191	-111.949	92	-107.359	180	-95.4309
172	-122.357	120	-116.972	109	-111.928	141	-107.056	35	-93.3435

**Table 5:** Best five compounds screened using RASPD from zinc data-base and further screening through iGEMDOCK.

ZINC id	B. E.
zinc_1158015	-129.626
zinc_793735	-127.627
zinc_13943005	-121.825
zinc_11790332	-117.637
zinc_8680620	-117.097

**Figure 2:**
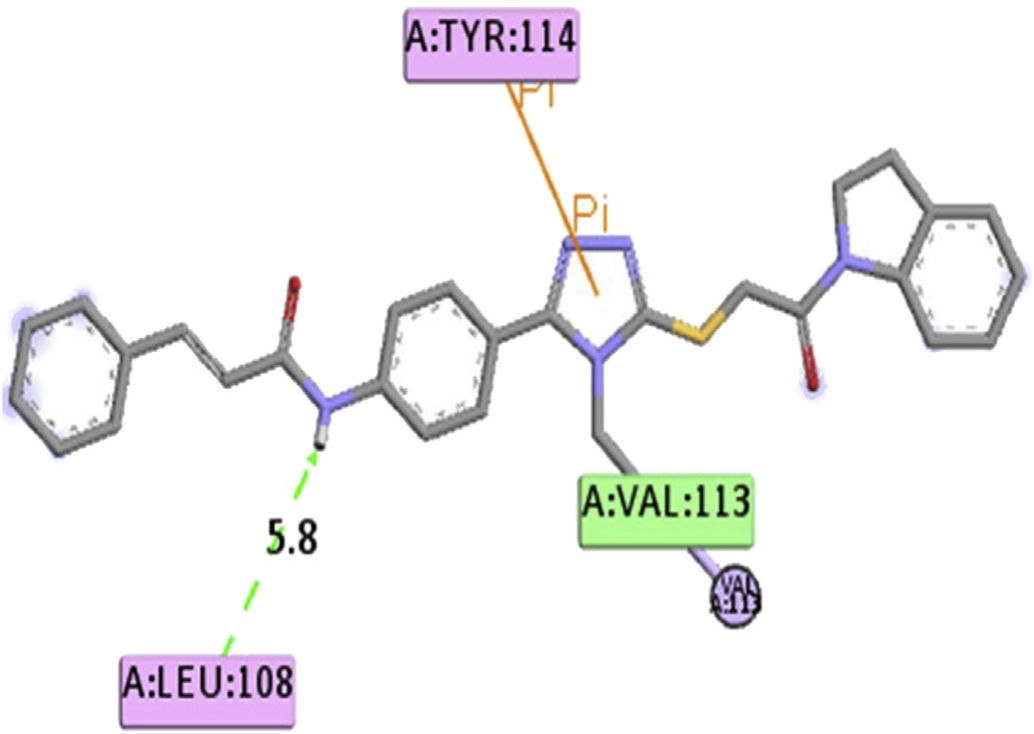
Screened drug molecule from zinc data base showed only oneπ-π and hydrogen bonding are represented as a stick model.

Binding energy of the molecules or compounds from the obtained from the zinc database were further studied using iGEMDOCK, it found that the molecules (zinc_1158015) showed minimum total binding energy but it was less than the designed best five compounds as in Table 4. This molecule showed only one π-π interaction with TYR-114 and two H. bond interaction with LEU108 and VAL113 in Figure 2.

Best five molecules from the designed library as in Table 4 as well as the screened molecules from zinc database as in Table 5 are taken for the prediction of physicochemical properties, lipophilicity, water solubility, pharmacokinetics, drug-likeness and bioactivity score as in Tables 6 and 7. All best five compounds follow the criteria of biological parameters. If a compound having GPCR ligand values > 0.00 is mostly likely to possess considerable biological activities, while ligand values -0.50 to 0.00 are expected to be moderately active and ligand values < -0.50, presumed to be inactive.

**Table 6:** Physiochemical properties, lipophilicity, water solubility, pharmacokinetics, drug-likeness, and bioactivity score of the designed best five compounds against nsp3 of CHIKV.

Property	Screened best five molecules from designed compounds against nsP3 of CHIKV				
	178	53	140	173	124
Log S	-3.54	-4.51	-3.75	-4.97	-3.75
Heavy atoms	28	31	29	30	29
MW (g/mol)	378.30	486.27	394.29	433.36	394.29
No. of rotational bonds	2	5	2	3	2
No. H-bond acceptors	6	6	7	7	7
Num. H-bond donors	1	1	2	1	2
Log Po/w (iLOGP)	1.61	2.69	1.51	2.57	1.09
GPCR ligand	-0.21	-0.18	-0.22	-0.00	-0.21
Lipinski	Yes, 0 violation	Yes,0 violation	Yes,1 violation: N or O > 10	Yes,0 violation	Yes,1 violation: N or O > 10
Log K_p_ in cm/s	-7.56	-7.59	-7.51	-6.55	-7.51
TPSA (Å^2^)	135.82	108.46	156.05	115.30	156.05
% ABS	62.14	71.58	55.16	69.22	55.16
Bioavailability Score	0.55	0.55	0.55	0.55	0.55
Synthetic accessibility	4.15	4.47	4.21	4.17	4.19
Physiochemical space for oral bioavailability

**Table 7:** Physicochemical properties, lipophilicity, water solubility, pharmacokinetics, drug-likeness, and bioactivity score of best five compounds from zinc database.

Physicochemical properties	Best five molecules from ZINC database against nsP3 of CHIKV				
	ZINC13943005	ZINC08680620	ZINC11790332	ZINC00793735	ZINC01158015
Log S	-5.74	-4.96	-4.99	-5.03	-5.51
Heavy atoms	36	33	36	33	36
MW (g/mol)	486.65	472.51	496.62	473.49	495.60
No. of rotational bonds	17	6	15	6	9
No. H-bond acceptors	2	6	5	7	4
Num. H-bond donors	4	1	2	1	1
Log Po/w (iLOGP)	3.95	3.48	3.84	3.34	3.84
GPCR ligand	0.24	-0.11	0.05	-0.17	-0.47
Lipinski	Yes; 0 violation	Yes; 0 violation	Yes; 0 violation	Yes; 0 violation	Yes; 0 violation
Log K_p_ in cm/s	-5.17	-6.78	-6.16	-6.71	-6.26
TPSA (Å^2^)	83.98	83.18	101.55	96.07	105.42
% ABS	80.02	80.30	73.96	75.85	72.63
Bioavailability Score	0.55	0.55	0.55	0.55	0.55
Synthetic accessibility	3.26	3.57	4.21	3.54	3.79
Physiochemical space for oral bioavailability					

For the prediction of acute toxicity, the adverse effects of a compound may result due to one or more than one time exposure. In present work, authors have determined the median lethal dose (LD_50_) of top five hit from the designed library (CMPD178, 53, 140, 173 & 124 as in Table 8) via four types of administration: oral, intravenous, intraperitoneal and subcutaneous. LD_50_ is the amount of molecule, can causes the death or kill the 50% of test animal. Therefore, the toxicologists can use different animals but rats and mice are usually considered for the study. It is expressed per 100 g of the body weight of the small animals.

**Table 8:** Rat acute toxicity and acute rodent toxicity was calculated from top five hit screened compound (CMPD178, 53, 140, 173 & 124).

C. No.	Rat acute toxicity (mg/kg)				Acute Rodent Toxicity			
	Rat IP LD_50_ (in AD)	Rat IV LD_50_ (in AD)	Rat Oral LD_50_ (in AD)	Rat SC LD_50_ (in AD)	Rat IP LD_50_ (in AD)	Rat IV LD_50_ (in AD)	Rat Oral LD_50_ (in AD)	Rat SC LD_50_ (in AD)
178	572,200	326,500	840,700	1300,000	Class 5	Class 5	Class 4	Class 5
53	608,000	156,400	875,300	1815,000 Out of AD	Class 5	Class 4	Class 4	Class 5 Out of AD
140	977,800	433,300	1095,000	952,100 Out of AD	Class 5	Class 5	Class 4	Class 4 Out of AD
173	633,500	458,800	146,900 Out of AD	1119,000	Class 5	Class 5	Class 3 Out of AD	Class 5
124	949,600	390,000	1312,000	989,300	Class 5	Class 5	Class 4	Class 4

Note: Where, in AD meaning the compound falls in applicability domain of models while out of AD means the compound is out of applicability domain of models.

**Figure 3:**
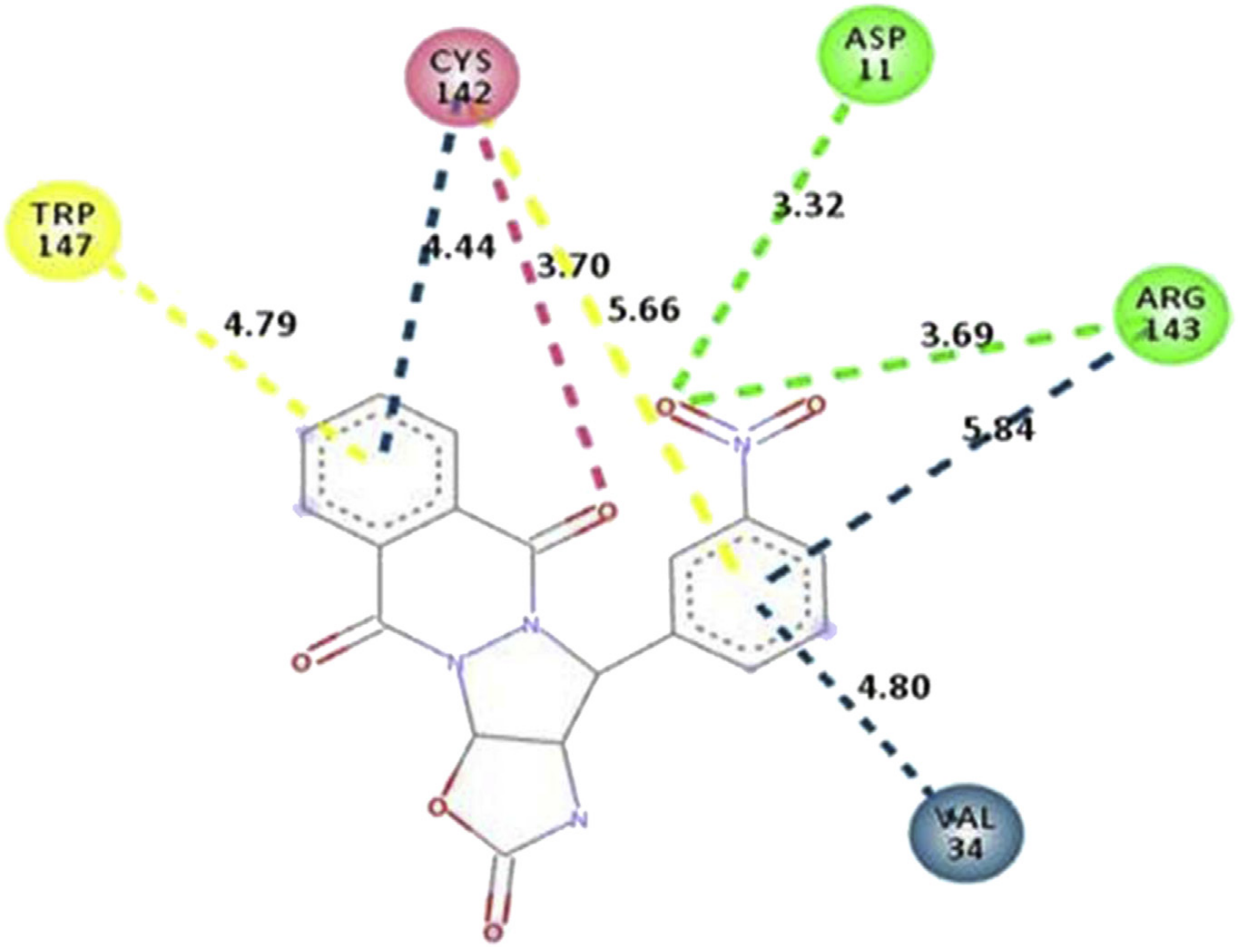
Interaction of CMPD178 drug molecule onto the active site of APR ligand bonded to 3GPO.

The active site of receptor is a binding pocket due to hydrogen bonding, hydrophobic interactions. Screened compounds showed the promising antiviral activity against nsp3 of CHIKV based on binding energy. Molecular docking method was used for predicting the binding energy of newly formed drug-target complex. In this study, best five compounds from designed library were docked with active site of nsp3 of CHIKV using Pardock to elucidate their molecular interactions as in Table 9 and Figure 3.

**Table 9:** Actual molecular docking results of CMPD178 drug molecule onto active site of APR ligand and also represented their interactions with distance analysis.

CMPD	Number of H-bonding	Interacted residue with distance (Å)
178	8	ASP11-O (8) = 3.32, ARG143-O (8) = 3.69, ARG143-r1 = 5.84, VAL34-r2 = 4.80, TRP147-R1=4.79, CYS142-r1=4.44, CYS142-r2=5.66, CYS142-O=3.70

**Figure 4:**
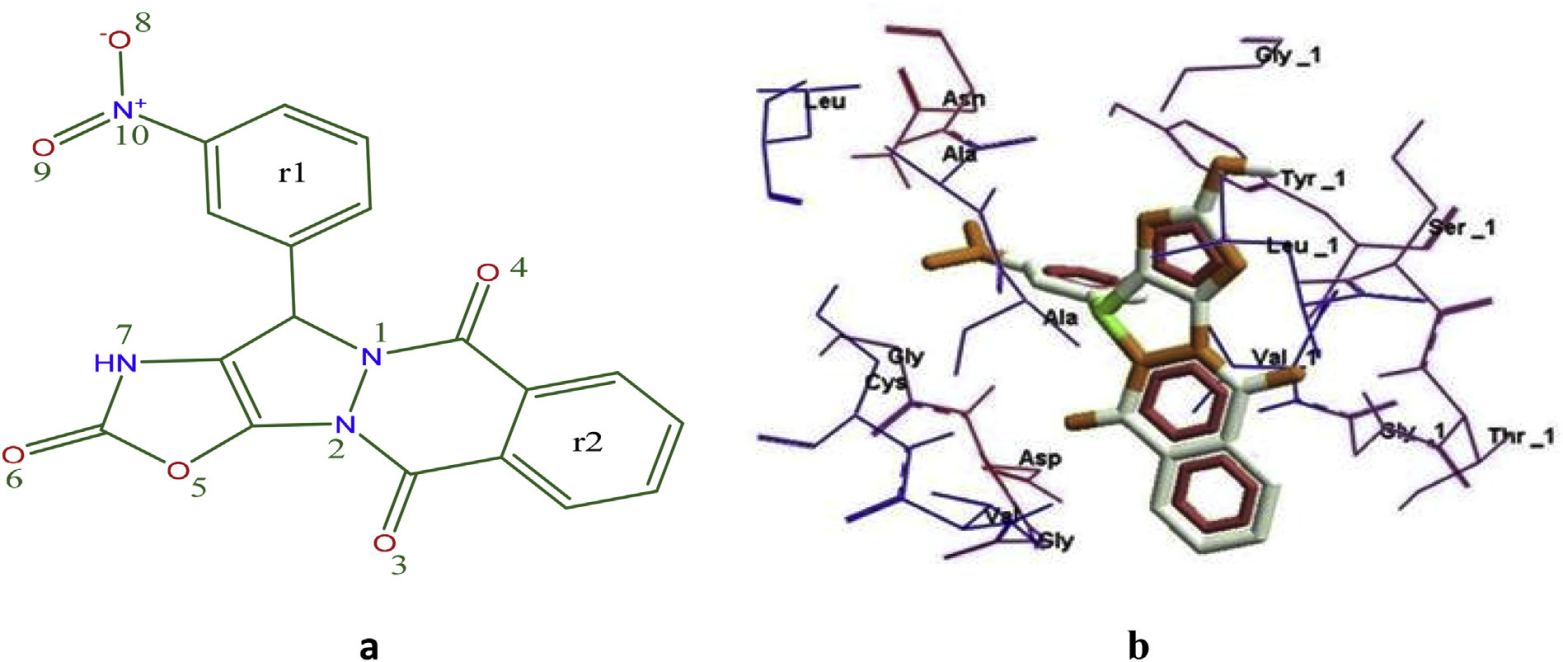
(a) 2D representation of CMPD178 drug molecules docked into the active site of the nsp3 of CHIKV; (b) Pose view of drug molecules inside the cavity of nsp3 of CHIKV.

The best docking pose for each ligand was also recorded for better results. This analysis was showed that molecules fit to bind in the cavity of nsp3 of CHIKV and by forming a stable d-t complex. As is evident from Figure 4a, b, drug interacts with nsp3 protease of CHIKV forming a most stable complex establishing hydrogen bond interactions with their minimum distance. The insight of various other residues are present in d-t complex interaction is depicted in 2D plot and these residues are play key role in the formation of stable d-t complex. The docking results were further evaluated in terms of RMSD value and binding free energy through MM-GBSA protocol.

### 3.2 MD simulation of target protein & drug-target complexes

**Figure 5:**
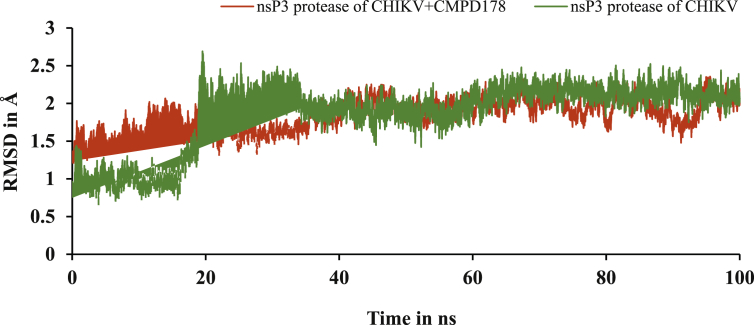
The RMSD plot of nsP3 of CHIKV with and without CMPD178 during MD simulations.

The AMBER18 program was used for MD Simulation to study the stability and flexibility of the nsp3 of CHIKV with and without CMPD178 receptor and its complex using different trajectories like RMSD, RMSF and hydrogen bond. RMSD plot showed that most of the complex system was relatively stable within 1–2.5 Å for 50–100 ns simulation time as in Figure 5.

**Figure 6:**
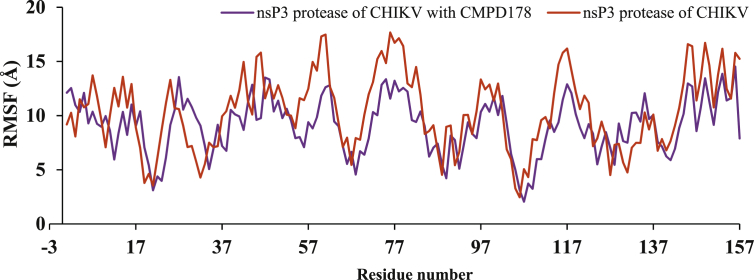
RMSF plot of nsp3 of CHIKV with and without CMPD178 for 100 ns.

**Figure 7:**
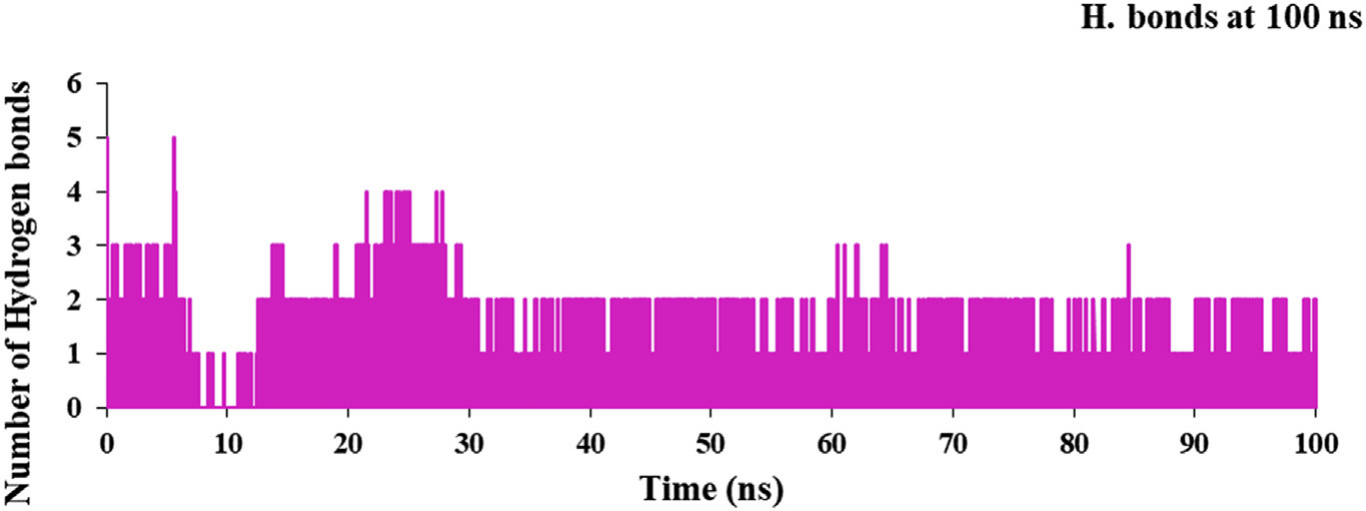
Hydrogen bond plot of nsP3 of CHIKV with and without CMPD178.

RMSF plot was used to understand the flexibility of the nsp3 of CHIKV with and without CMPD178 as in Figure 6. Less fluctuation are observed in the complex in comparison of the nsp3 of CHIKV alone. Further, the hydrogen bond plot and analysis for the complex of nsp3 of CHIKV-178 are given in Figure 7 and Table 10 respectively. It was used to find the existence of HBs between a donor and acceptor, % occupancy and angle during the simulations.

**Table 10:** Hydrogen bond analysis for the complex of nsp3 of CHIKV with CMPD178.

S. No.	Acceptor	Donor H	Donor N	Occupancy	Avg. Dist.	Avg. Ang.
1.	GLY_33@O	DRG_157@H5	DRG_157@N2	0.3521	2.8440	153.0793
2.	DRG_157@O2	ARG_143@HE	ARG_143@NE	0.0404	2.8489	153.1615
3.	DRG_157@O3	ILE_12@H	ILE_12@N	0.0305	2.8813	157.9834
4.	DRG_157@O2	ARG_143@HH21	ARG_143@NH2	0.0224	2.8559	146.3615
5.	DRG_157@O3	LYS_40@HZ2	LYS_40@NZ	0.0203	2.8501	153.3168
6.	DRG_157@O3	LYS_40@HZ3	LYS_40@NZ	0.0182	2.8562	153.6067
7.	DRG_157@O3	LYS_40@HZ1	LYS_40@NZ	0.0171	2.8530	153.5234
8.	DRG_157@O4	ILE_12@H	ILE_12@N	0.0058	2.9155	160.9870
9.	DRG_157@O4	LEU_108@H	LEU_108@N	0.0050	2.9110	150.9374
10.	TYR_141@O	DRG_157@H5	DRG_157@N2	0.0044	2.8748	156.1100

Residues ASP11, ARG143, THR111, LEU108, GLY112, ALA23 and VAL113 are present in the active site of nsP3 of CHIKV and showed noteworthy fluctuations compared to other native residues. The total numbers of average HBs are formed during MD simulations were predicted. The analysis to find the maximum number of hydrogen bonds is done and suggested maximum of 5 intermolecular hydrogen bonds. Average number of HBs for different donor-acceptor average distance cutoffs is 2.84 (strong bonding) with larger average angle. It was found that HBs between drug molecules and residues are GLY_33@O with donor H & N of DRG_157 as in Table 10. It was assume that formed HBs have distance between accepter residue O atom in the backbone with donor H & N in the drug molecules showed shorter distance (2.84 Å) and the angle of N–H–O is 153.07° with 35.21 % occupancy at 300K for 100 ns simulations time observed.

**Figure 8:**
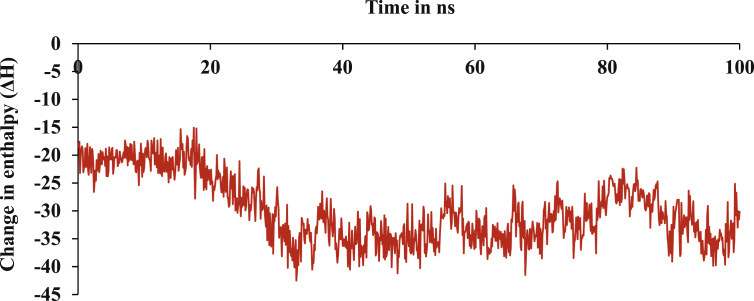
Change in enthalpy of the formation of complex of nsp3 of CHIKV with CMPD178 by MM-GBSA for the MD simulation 100 ns.

Binding free energies was calculated of drug (CMPD178), target and drug-target complex using MM-GBSA methods are shown in Table 11. Change in enthalpy (ΔH) as in Figure 8, differences of (drug-target complex) with target and drug was found to be -24.28 kcal/mol.

**Table 11:** The calculated change in enthalpy for drug-target complex, target and drug (kcal/mol).

Energy Component	d-t complex	t (nsp3 of CHIKV)	d (CMPD178)	Differences {d-t complex – (t + d)}	
	Average	Average	Average	Average	Std. Err. of Mean
BOND	475.79	464.27	11.51	-0.00	0.00
ANGLE	1265.19	1226.84	38.34	-0.00	0.00
DIHED	1978.12	1939.84	38.28	-0.00	0.00
VDWAALS	-1190.90	-1153.08	-5.19	-32.62	0.01
EEL	-11166.53	-11093.26	-58.25	-15.07	0.02
1-4 VDW	557.8752	543.22	14.65	-0.00	0.00
1-4 EEL	6673.11	6597.49	75.62	-0.00	0.00
EGB	-2422.47	-2419.33	-30.13	27.02	0.02
ESURF	42.78	43.07	3.36	-3.65	0.07
ΔG_gas_	-12357.43	-12246.34	-63.45	-47.63	0.02
ΔG_solv_	-2379.68	-2376.25	-26.77	23.34	0.02
ΔH_total_	-14737.12	-14622.60	-90.23	-24.28	0.01

Change in free energy (**ΔG**) was determined by calculating change in entropy (ΔS) and change in enthalpy (ΔH) for the formation of complex between nsp3 of CHIKV and CMPD178. For any spontaneous process or reaction, the change in free energy should be negative. TΔS was calculated for the complex system is -11.28 kcal/mol as in Table 12. ΔG for the binding of the complex between nsp3 of CHIKV and CMPD178 comes out to be -13.01 kcal/mol by using Eq. (10).

(10) **ΔG = ΔH – TΔS**

**Table 12:** Calculated change in entropy by using Quasi-harmonic approximation with CPPTRAJ at 298.15 K for 100 ns.

Systems	Translational	Rotational	Vibrational	Total
T∗Complex	16.41	16.52	2721.55	2754.49
T∗Receptor	16.39	16.50	2678.32	2711.22
T∗Ligand	13.01	10.66	30.87	54.54
TΔS	-12.99	-10.64	12.35	-11.28

### 3.3 Temperature dependent MD simulations (tdMD) and MM-GBSA

**Figure 9:**
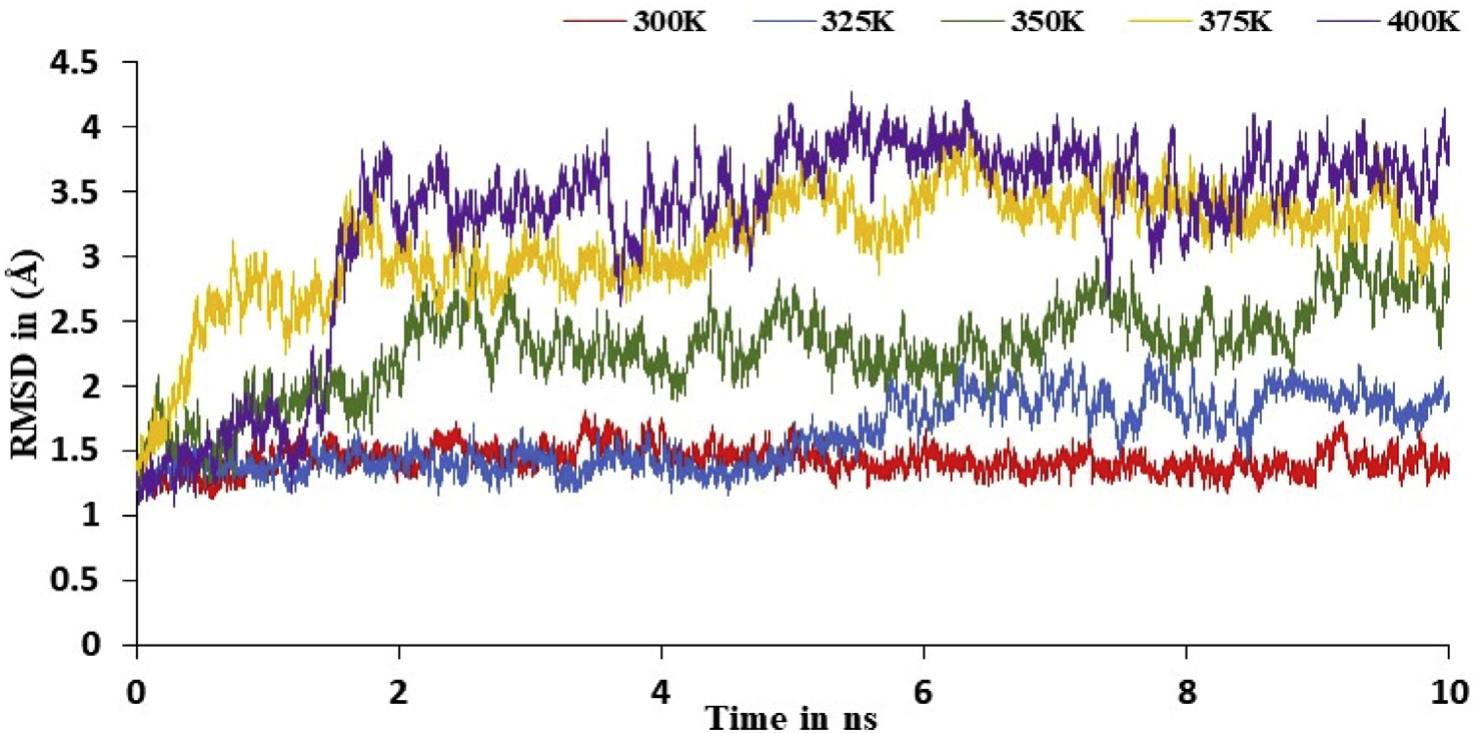
The RMSD plot of drug-target complex at variable temperature like 300, 325, 350, 375 and 400K.

In the literature, authors were explained MD simulation of backbone of nsp3 of CHIKV with and without CMPD178 (target protein and drug-target complex) at 300K (isothermally) and 1 atm pressure. Herein, based on David and Konrad approximation, authors varied the temperature from 300 to 400K (non-isothermally) and 1 atm pressure for MD simulation of drug-target complex. The system minimization, heating, and equilibration were carried out in the same manner used for the optimization of drug-target complex described above. In this way tdMD simulations were performed for 10 ns at 325, 350, 375 and 400K and the RMSD trajectories are given in Figure 9.

**Figure 10:**
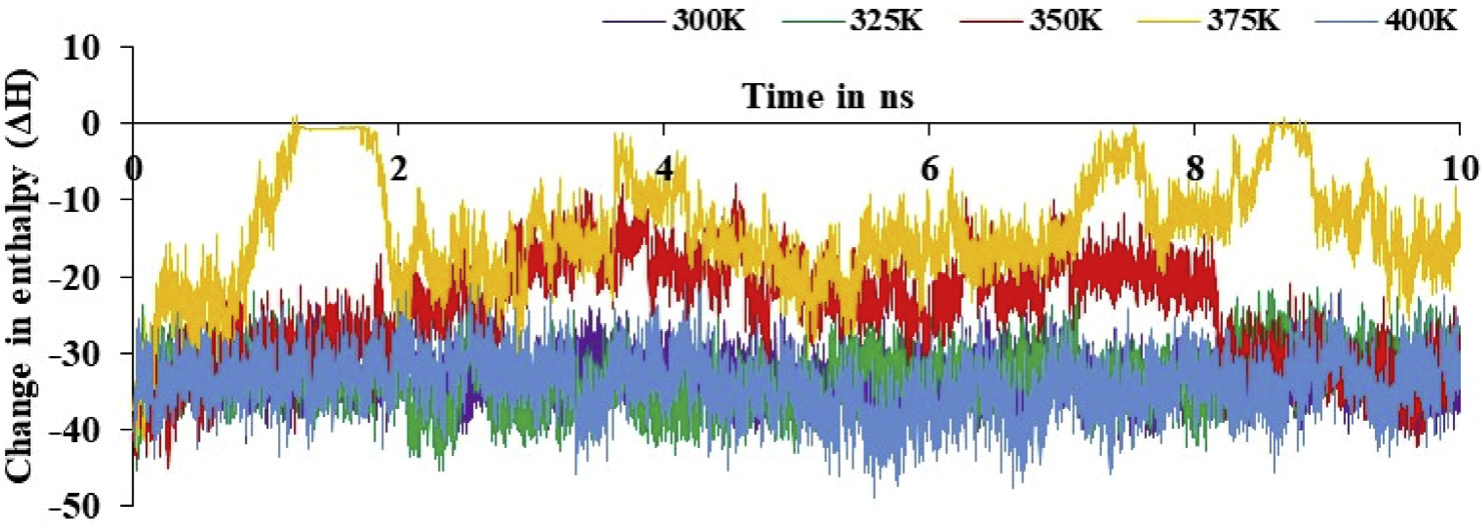
The change in relative enthalpy of variable temperature for 10 ns simulations time using MM-GBSA method.

**Figure 11:**
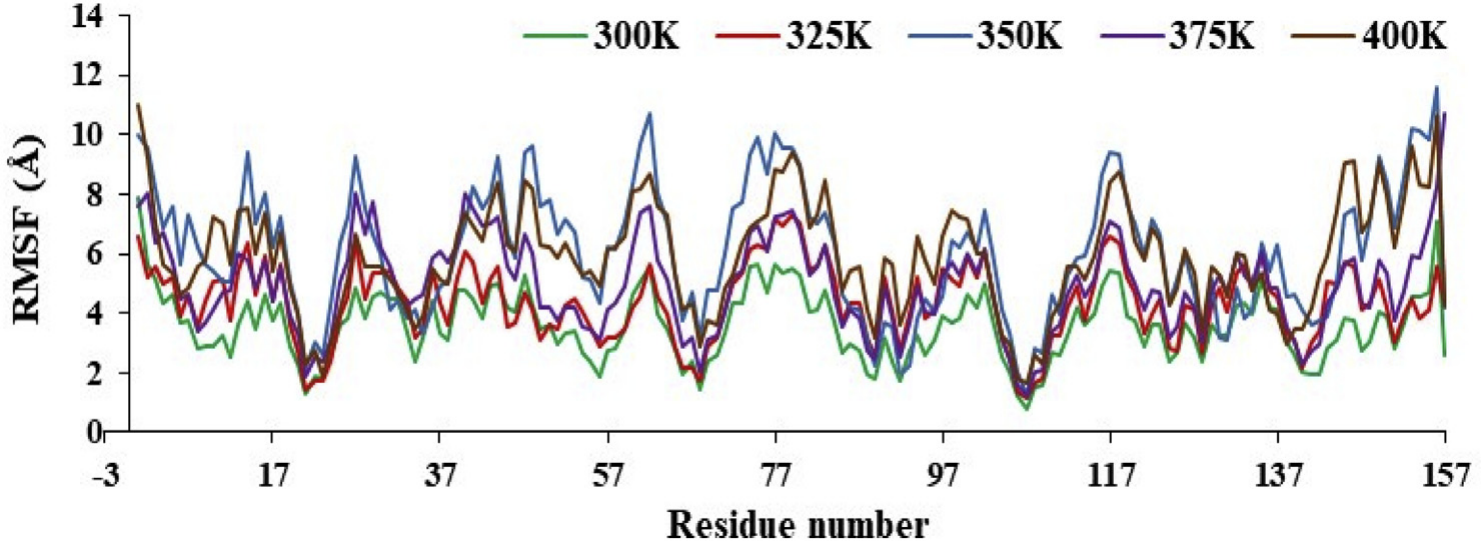
RMSF plot of nsp3 of CHIKV with CMPD178 complex for 100 ns at 300, 325, 350, 375 and 400K.

The simulations and change in relative enthalpy energy results confirmed that actual stability of system at 300 and 400K in Figure 10 and Table 13. A total of 10000 snapshots were taken in a 10 ns MD simulations time to calculate the binding free energy difference using Eq. (1). Further, RMSF curve for target and its complex with 178 drug molecules was studied at 10 ns at 300K, 325K, 350K, 375K and 400K as in Figure 11.

**Table 13:** The calculated binding free energies drug-target complex, target, drug and differences of drug-target complex with target and drug (kcal/mol) at different temperature.

Energy Component	Differences at 300K		Differences at 325K		Differences at 350K		Differences at 375K		Differences at 400K	
	Average	Std. Err. of Mean	Average	Std. Err. of Mean	Average	Std. Err. of Mean	Average	Std. Err. of Mean	Average	Std. Err. of Mean
BOND	-0.00	0.00	-0.00	0.00	-0.00	0.00	-0.00	0.00	0.00	0.00
ANGLE	-0.00	0.00	0.00	0.00	-0.00	0.00	-0.00	0.00	0.00	0.00
DIHED	0.00	0.00	0.00	0.00	-0.00	0.00	0.00	0.00	0.00	0.00
VDWAALS	-44.15	0.02	-43.54	0.03	-34.43	0.07	-19.92	0.09	-44.86	0.04
EEL	-15.92	0.05	-12.91	0.07	-9.88	0.08	-7.12	0.08	-12.42	0.07
1-4 VDW	0.00	0.00	0.00	0.00	0.00	0.00	0.00	0.00	0.00	0.00
1-4 EEL	-0.00	0.00	0.00	0.00	-0.00	0.00	-0.00	0.00	0.00	0.00
EGB	31.15	0.05	27.67	0.05	23.57	0.09	15.67	0.09	27.76	0.07
ESURF	-4.36	0.00	-4.41	0.00	-3.91	0.00	-2.75	0.00	-4.68	0.00
ΔG_gas_	-60.08	0.06	-56.46	0.08	-44.32	0.14	-27.04	0.14	-57.28	0.09
ΔG_solv_	26.78	0.04	23.25	0.05	19.65	0.09	12.92	0.08	23.08	0.07
ΔH_total_	-33.29	0.02	-33.20	0.03	-24.66	0.06	-14.11	0.07	-34.20	0.03

**Figure 12:**
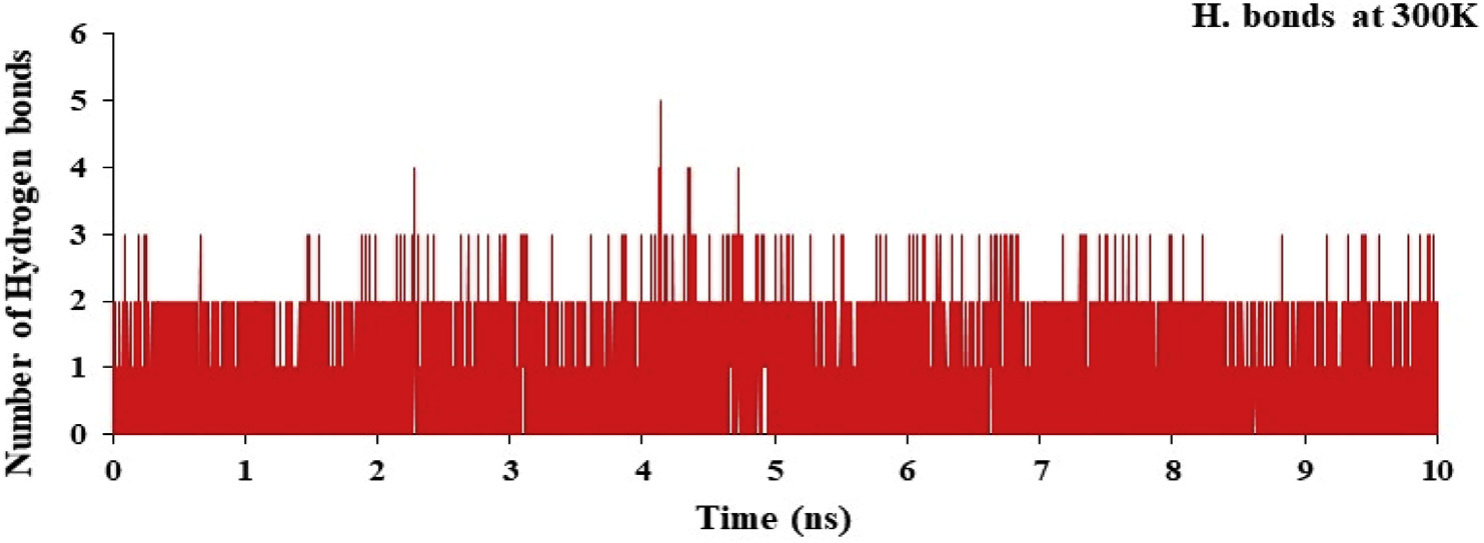
Hydrogen bond plot of nsp3 of CHIKV with CMPD178 for 10 ns at 300K.

**Figure 13:**
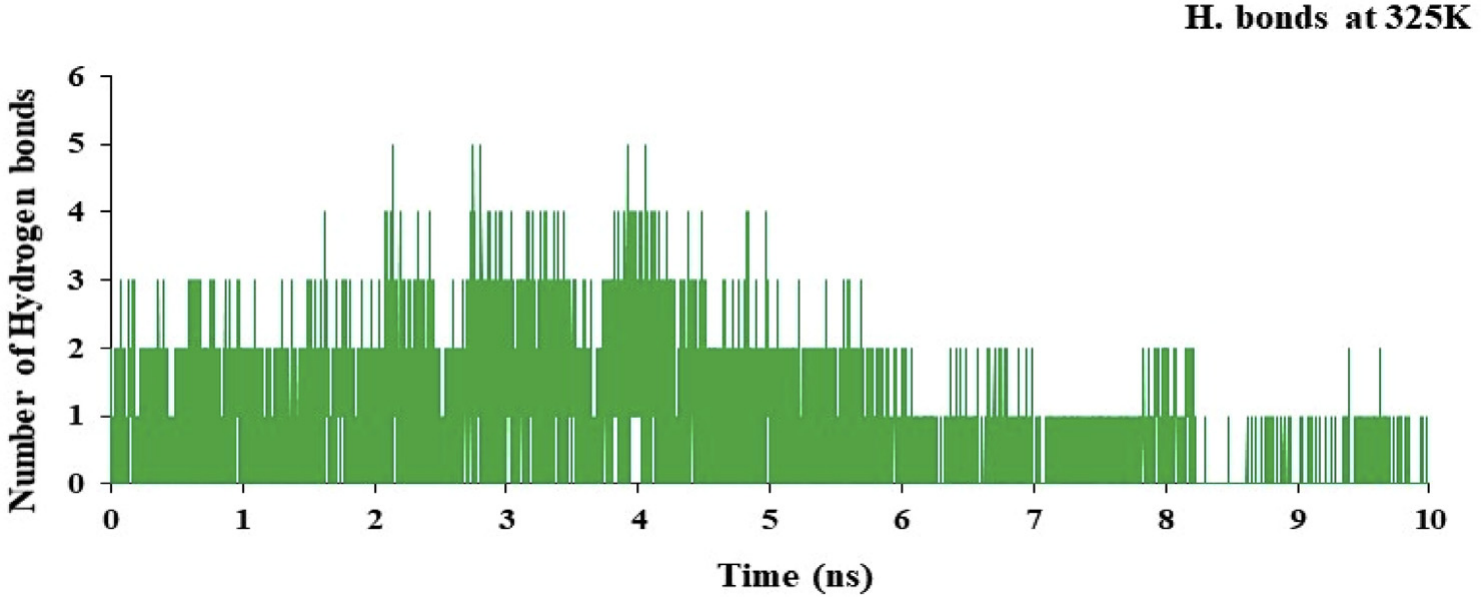
Hydrogen bond plot of nsP3 of CHIKV with CMPD178 for 10 ns at 325K.

**Figure 14:**
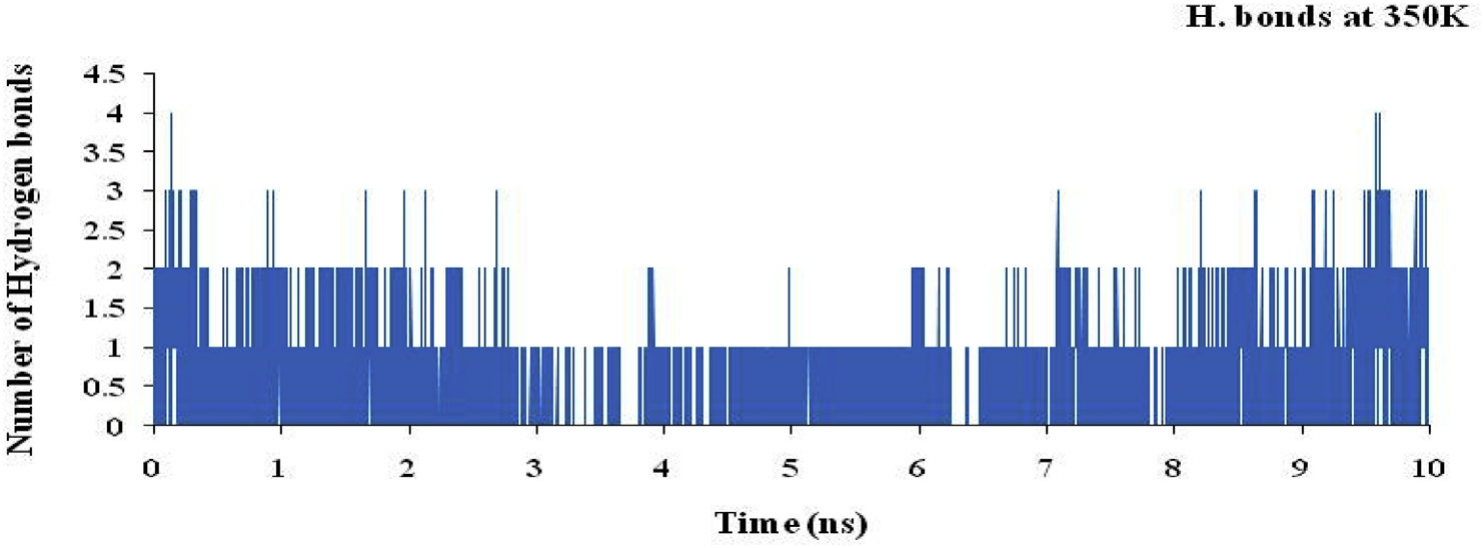
Hydrogen bond plot of nsp3 of CHIKV with CMPD178 for 10 ns at 350K.

**Figure 15:**
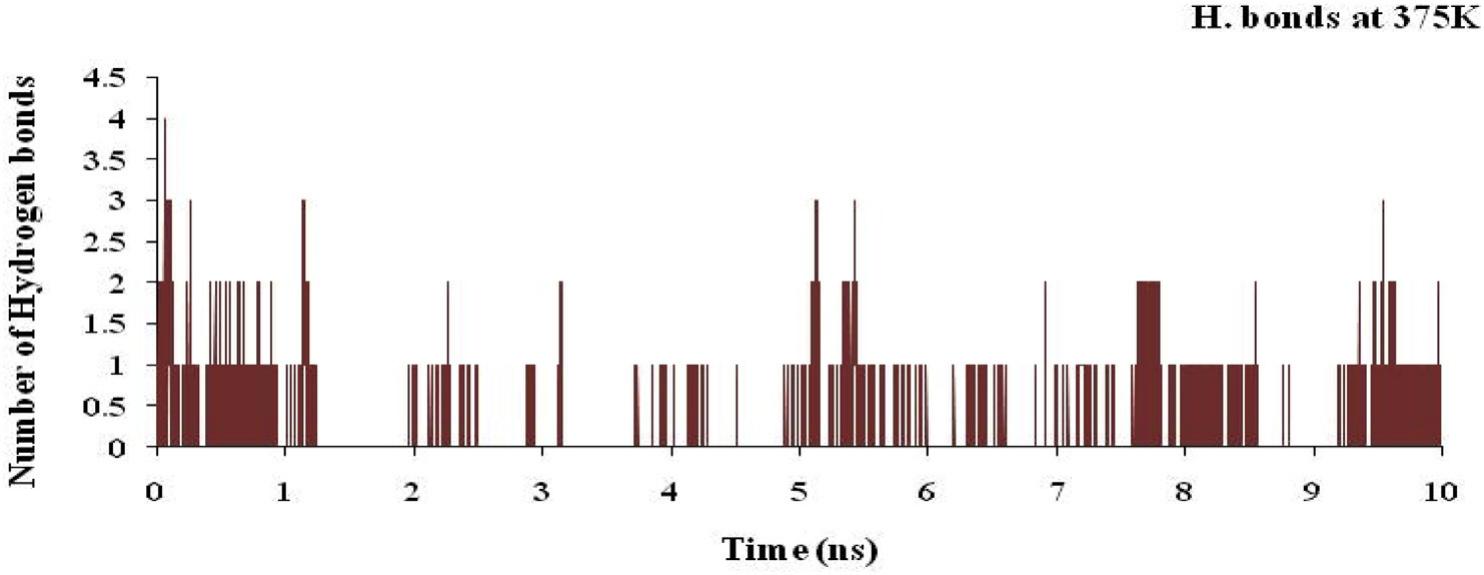
Hydrogen bond plot of nsp3 of CHIKV with CMPD178 for 10 ns at 375K.

**Figure 16:**
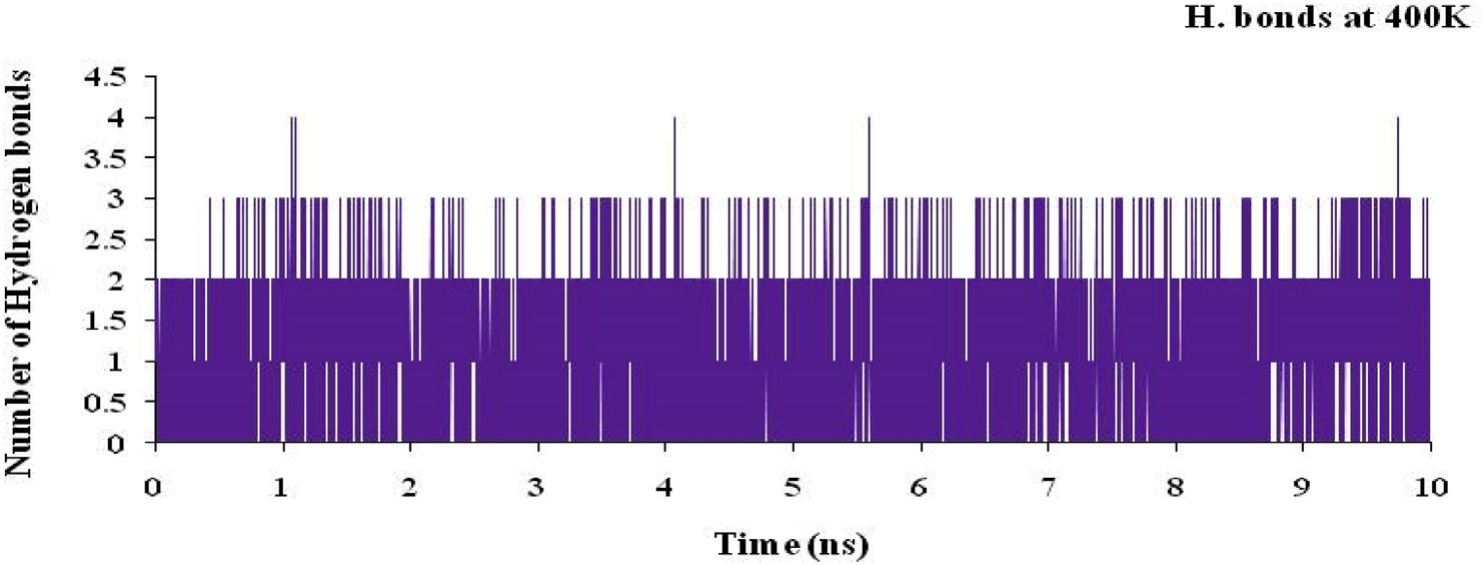
Hydrogen bond plot of nsP3 of CHIKV with CMPD178 for 10 ns at 400K.

The hydrogen bond plot for the complex of nsp3 of CHIKV-178 for 10 ns at 300, 325, 350, 375 and 400K was studied and maximum number of hydrogen bonds for the complex at 300, 325, 350, 375 and 400K are 5, 5, 4, 4 and 4 respectively and shown in Figures 12, 13, 14, 15, and 16 respectively. It means on increasing the temperature, number of hydrogen bonds decreases. Further, hydrogen bond analysis for the complex of nsp3 of CHIKV-178 for 10 ns at 300, 325, 350, 375 and 400K was given in Tables 14, 15, 16, 17, and 18 respectively. Prediction of the structural stability of intermolecular hydrogen bonds and total number of hydrogen bonds formed with nsp3 of CHIKV. MD simulation of the drug-target complex is used to study the stability during the trajectory period. Hydrogen bond profiles between the selected drugs and nsp3 of CHIKV were calculated using the AMBER18. This analysis revealed that average hydrogen bonds are formed during the simulations period sharing four to five hydrogen bonds with GLY33, ARG143, ILE12, LYS40, LEU108 and these five hydrogen bonds showed poor hydrogen bond interactions with weak fractions of time at 300K for 100 ns time period in Figure 7 and Table 10. The same pattern was also observed in the case of variable temperature but at 400K, TYR141 showed maximum fraction of time in Figure 16 and Table 18.

**Table 14:** Hydrogen bond analysis for the complex of nsp3 of CHIKV-178 for 10 ns at 300K.

S. No.	Acceptor	Donor H	Donor N	Occupancy	Avg. Dist.	Avg. Ang.
1.	DRG_157@O3	ILE_12@H	ILE_12@N	0.4970	2.8767	155.774
2.	DRG_157@O4	LEU_108@H	LEU_108@N	0.1362	2.8956	151.922
3.	DRG_157@O5	LEU_108@H	LEU_108@N	0.0919	2.9016	152.489
4.	TYR_141@O	DRG_157@H5	DRG_157@N2	0.0877	2.8909	163.204
5.	CYS_142@O	DRG_157@H5	DRG_157@N2	0.0469	2.8640	143.1449
6.	DRG_157@O1	ARG_143@HH21	ARG_143@NH2	0.0045	2.8503	150.8655
7.	DRG_157@O1	ARG_143@HE	ARG_143@NE	0.0037	2.8677	150.7055
8.	DRG_157@O5	THR_111@HG1	THR_111@OG1	0.0030	2.8607	155.6787
9.	DRG_157@O4	THR_111@HG1	THR_111@OG1	0.0018	2.8699	149.1284
10.	DRG_157@O3	ARG_143@HH11	ARG_143@NH1	0.0004	2.9278	142.3369

**Table 15:** Hydrogen bond analysis for the complex of nsp3 of CHIKV-178 for 10 ns at 325K.

S. No.	Acceptor	Donor H	Donor N	Occupancy	Avg. Dist.	Avg. Ang.
1.	TYR_141@O	DRG_157@H5	DRG_157@N2	0.3578	2.8639	158.1934
2.	DRG_157@O3	ILE_12@H	ILE_12@N	0.1336	2.8855	153.9108
3.	DRG_157@O1	ARG_143@HH21	ARG_143@NH2	0.0751	2.8547	151.3636
4.	DRG_157@O5	LEU_108@H	LEU_108@N	0.0743	2.9011	155.9742
5.	DRG_157@O1	ARG_143@HE	ARG_143@NE	0.0584	2.8684	151.7363
6.	DRG_157@O4	LEU_108@H	LEU_108@N	0.0551	2.9004	155.4182
7.	DRG_157@O2	VAL_113@H	VAL_113@N	0.0150	2.9026	161.3469
8.	DRG_157@O2	THR_111@HG1	THR_111@OG1	0.0149	2.7488	161.4391
9.	CYS_142@O	DRG_157@H5	DRG_157@N2	0.0052	2.8668	143.4847
10.	DRG_157@O5	THR_111@HG1	THR_111@OG1	0.0024	2.8525	153.3075

**Table 16:** Hydrogen bond analysis for the complex of nsp3 of CHIKV-178 for 10 ns at 350K.

S. No.	Acceptor	Donor H	Donor N	Occupancy	Avg. Dist.	Avg. Ang.
1.	DRG_157@O1	ASP_11@H	ASP_11@N	0.1016	2.8440	156.8376
2.	ARG_143@O	DRG_157@H5	DRG_157@N2	0.0812	2.8326	156.0430
3.	TYR_141@O	DRG_157@H5	DRG_157@N2	0.0695	2.8540	157.8781
4.	CYS_142@O	DRG_157@H5	DRG_157@N2	0.0673	2.8478	152.9286
5.	ARG_10@O	DRG_157@H5	DRG_157@N2	0.0666	2.8486	156.5747
6.	DRG_157@O4	LEU_108@H	LEU_108@N	0.0298	2.8919	152.5300
7.	DRG_157@O5	LEU_108@H	LEU_108@N	0.0284	2.9007	154.8072
8.	VAL_34@O	DRG_157@H5	DRG_157@N2	0.0259	2.8287	150.2875
9.	DRG_157@O3	ARG_143@HH11	ARG_143@NH1	0.0168	2.8456	157.0241
10.	DRG_157@O1	ARG_143@HH11	ARG_143@NH1	0.0167	2.8296	153.6600

**Table 17:** Hydrogen bond analysis for the complex of nsp3 of CHIKV-178 for 10 ns at 375K.

S. No.	Acceptor	Donor H	Donor N	Occupancy	Avg. Dist.	Avg. Ang.
1.	DRG_157@O2	ASN_25@HD21	ASN_25@ND2	0.0226	2.8542	159.5127
2.	DRG_157@O2	ARG_27@HE	ARG_27@NE	0.0224	2.8471	153.4673
3.	DRG_157@O2	ARG_27@HH11	ARG_27@NH1	0.0171	2.8360	150.1817
4.	DRG_157@O1	ILE_12@H	ILE_12@N	0.0170	2.8755	159.2632
5.	DRG_157@O2	LEU_29@H	LEU_29@N	0.0144	2.8779	157.7655
6.	DRG_157@O3	ILE_12@H	ILE_12@N	0.0107	2.8717	157.2507
7.	DRG_157@O1	LYS_40@HZ1	LYS_40@NZ	0.0081	2.8181	153.6666
8.	DRG_157@O1	LYS_40@HZ3	LYS_40@NZ	0.0066	2.8417	154.3329
9.	TYR_141@O	DRG_157@H5	DRG_157@N2	0.0062	2.8734	161.1651
10.	DRG_157@O2	TYR_39@HH	TYR_39@OH	0.0060	2.7157	157.5764

**Table 18:** Hydrogen bond analysis for the complex of nsp3 of CHIKV-178 for 10 ns at 400K.

S. No.	Acceptor	Donor H	Donor N	Occupancy	Avg. Dist.	Avg. Ang.
1.	TYR_141@O	DRG_157@H5	DRG_157@N2	0.6688	2.8431	158.3383
2.	DRG_157@O2	THR_111@HG1	THR_111@OG1	0.1200	2.7640	155.7204
3.	DRG_157@O3	ILE_12@H	ILE_12@N	0.1072	2.8922	155.5073
4.	DRG_157@O5	LEU_108@H	LEU_108@N	0.0505	2.8993	156.8353
5.	DRG_157@O4	LEU_108@H	LEU_108@N	0.0499	2.8966	157.0258
6.	DRG_157@O3	ARG_143@H	ARG_143@N	0.0402	2.8818	143.2893
7.	DRG_157@O1	VAL_34@H	VAL_34@N	0.0288	2.8797	161.2791
8.	DRG_157@O1	ARG_143@HH11	ARG_143@NH1	0.0285	2.8454	153.7032
9.	DRG_157@O1	ARG_143@HH21	ARG_143@NH2	0.0035	2.8205	155.3732
10.	DRG_157@O1	ARG_143@HE	ARG_143@NE	0.0029	2.8604	148.6303

### 3.4 DFT calculations

DFT calculations of best five compounds have been performed and frontier molecular orbitals taken as in Table 19. HOMO-LUMO energy gap plays an important role in stabilizing the interactions between compound and nsp3 of CHIKV. By using energy values of HOMO and LUMO for top five screened hit drug molecule from designed library to calculated μ, χ, η and ω by using Eqs. (6), (7), (8), and (9). Table 20 summarizes the HOMO, LUMO and HOMO-LUMO energy gaps (ΔE) of top five hit drug molecules calculated at DFT level in the B3LYP/6-311G∗ basis set.

**Table 19:** Graphical representation of HOMO and LUMO of best five compounds.

CMPD	HOMO	LUMO	CMPD	HOMO	LUMO
178			173		
53			124		
140

**Table 20:** Energy value of E_HOMO_, E_LUMO_, ΔE, ɳ, χ, μ and ω of the best five compounds (178, 53, 140, 173 &124).

CMPD	E_HOMO_	E_LUMO_	ΔE	μ	ɳ	Χ	Ω
178	-0.16947	-0.13643	-0.03304	-0.15295	0.01652	0.15295	0.70805
53	-0.22716	-0.08723	-0.13993	-0.15719	0.01796	0.15719	0.17658
140	-0.18815	-0.14363	-0.04452	-0.16589	0.02226	0.16589	0.61814
173	-0.23145	-0.09110	-0.14035	-0.16127	0.07017	0.16127	0.18531
124	-0.23801	-0.11537	-0.12264	-0.17669	0.06132	0.17669	0.25456

The energy different between HOMO and LUMO is used to understand the chemical reactivity and kinetic of molecules. If a compound has small energy gap indicates more polarizable and therefore have high chemical reactivity and termed as soft molecule.

Global term is a primary descriptor for the chemical reactivity of compounds. Chemical hardness is a measure to study the stability of compound. It explains the resistance towards polarization of the electron cloud under small perturbation. Chemical potential is a form of energy and can be absorbed or released on changing the number of the species in a chemical reaction. Larger the value of electronegativity indicates more the attractiveness for electrons. Electrophilicity is a measure for the energy stabilization of compound. It is used to understand the the reactivity of compounds involved in chemical reactions.

## 4 Conclusion

In the present work, finding the promising candidate against nsp3 of CHIKV was explored via screening, docking, MD simulations, MM-GBSA calculation. A library of compounds is created based on the product obtained in one pot three component reaction. Then, the compounds were subjected to docking and bioactive score. Further, the results of screened compounds were compared with results of the compounds based on the compounds obtained from the RASPD. Then, nsp3 of CHIKV with and without CMPD178 were studied using MD simulations for 100 ns and change in binding energy is determined by MM-GBSA method. ΔG for the formation of complex was found to be -13.01 kcal/mol. Subsequently, the effect of temperature was studied for the formation of the complex between the nsp3 of CHIKV and CMPD178 using the MD simulations. The RMSD values and fluctuation increased on increasing the temperature. Therefore, it is understood that the best inhibition is observed at 300K by the CMPD178.

## Declarations

### Author contribution statement

Durgesh Kumar: Performed the experiments; Analyzed and interpreted the data; Wrote the paper.

Mahendra Kumar Meena: Contributed reagents, materials, analysis tools or data; Wrote the paper.

Kamlesh Kumari, Prashant Singh: Conceived and designed the experiments; Wrote the paper.

Rajan Patel: Analyzed and interpreted the data.

Abhilash Jayaraj: Performed the experiments; Analyzed and interpreted the data.

### Funding statement

This research did not receive any specific grant from funding agencies in the public, commercial, or not-for-profit sectors.

### Competing interest statement

The authors declare no conflict of interest.

### Additional information

No additional information is available for this paper.
